# Impact of Molecular Profiling of Breast Cancer on the Rate of Locoregional Recurrence in Young Versus Old Female Patients

**DOI:** 10.7759/cureus.12438

**Published:** 2021-01-03

**Authors:** Hesham Soliman, Mohamed Abouelazayem, Mohamed Elkorety, Mohamed Akram Nouh, Eman M Touny, Hassan M Abdalla

**Affiliations:** 1 Department of General Surgery, Kings College NHS Foundation Trust, London, GBR; 2 Department of Surgical Oncology, National Cancer Institute, Cairo University, Cairo, EGY; 3 Department of General Surgery, St George’s University Hospitals NHS Foundation Trust, London, GBR; 4 Department of General Surgery, West Hertfordshire Hospitals NHS Trust, Watford, GBR; 5 Department of Pathology, National Cancer Institute, Cairo University, Cairo, EGY

**Keywords:** locoregional recurrence, breast cancer, molecular subtypes, triple negative, her2/neu-enriched, luminal

## Abstract

Background

Breast cancer (BC) is diverse regarding its natural history and treatment responses. The traditional histopathological classification is unable to confine this diverse clinical heterogeneity. Classically, prognosis and treatment response are influenced by factors including histological grade, lymph node status, and tumour size. Recently, research has diverted from histological classification towards molecular classification.

We aim to analyse the locoregional recurrence of breast cancer incidence following surgery across the different molecular subtypes as well as relation to age.

Materials and methods

Female patients diagnosed with a locoregional recurrence of breast carcinoma in 2012-2014 were identified from our centre histology department. We only included stage I-III patients who were previously treated with surgery achieving negative surgical margins and later developed locoregional recurrence during our study period. These patients were subdivided by age into old (≥40 years old) and young (<40 years old) groups according to their initial diagnosis age. Furthermore, they were categorised according to the molecular subtype of their primary tumour.

Results

Our study included 184 patients (124 designated to the old age group, 60 to the young age group). In the young group, recurrence occurred after a mean of 4.3 years and the range was one to 23 years, while in the old group, the mean was 3.8 years, and the range was one to 14 years. The most primary cancer subtype recorded was triple-negative (41.85%): 50 old patients and 27 young. Next was the Her-2/neu enriched subtype (27.72%): 35 old patients and 16 young, following this was luminal A subtype (21.19%): 27 old and 12 young. Last was the luminal B subtype (9.24%): 12 old patients and five young.

Conclusions

To conclude, in our series, the most common molecular subtype found in the recurrent cases was the luminal negative subtypes, with a relatively similar pattern across both age groups. The results of this study can be used as a basis for large prospective studies in our centre to further analyse the effect of molecular subtyping on the recurrence rates of BC.

## Introduction

Breast cancer (BC) is the most common cancer worldwide [1]. Roughly 250,000 new cases are diagnosed yearly in the United States, which represents 14% of all newly diagnosed cases [2]. While in the United Kingdom, more than 50,000 cases are diagnosed yearly, which accounts for 15% of yearly diagnosed cancers [3]. BC is considered diverse regarding disease progression as well as response to treatment [4]. Traditionally, BC prognosis was linked to factors as lymph node status, histological grade, and tumour size. The classic histological classification of BC is incapable of confining this diversity with regards to its clinical heterogeneity [5]. Surgery has always been central to the curative treatment of BC. Ongoing advances in adjuvant therapy have paved the way for a less radical approach [6].

Recently, BC research has diverted from the classical classification by histology towards molecular profiling and subtyping of BC [7]. Many randomized controlled trials examined the difference between locoregional recurrence rates for both modified radical mastectomy (MRM) and breast conservative surgery (BCS); the locoregional recurrence rates for BCS was slightly higher, the range in these studies was 3-20% and 2-14% for BCS and MRM respectively. The main etiologies for locoregional recurrence were inadequate surgical margins, poor planning or execution of adjuvant radiotherapy or a biologically aggressive tumour as triple-negative subtype which is thought to have a higher risk for locoregional recurrence than the luminal subtypes [8].

The impact of triple-negative BC on locoregional recurrence after BCS has been studied by many with conflicting reports. Some have stated no significant increase [9-10]. On the other hand, others have published a higher risk [11]. However, mutually these studies suggested that the slightly higher locoregional recurrence for triple-negative BC subtype than the luminal subtypes should not discourage BCS and adjuvant radiation for clinically suitable patients diagnosed with triple-negative BC [12].

The age of the patient has been regarded as an independent risk factor for the development of locoregional recurrence after surgical management of BC and is regarded as strong as margin affection for patients under the age of 40 years [13]. Age is the most important risk factor for the appearance of local relapse after preservation treatment, but not after mastectomy [14-15]. Even though age is the most important prognostic factor in the majority of studies, there is no justification for not carrying out BCS for young patients, as MRM did not improve the relapse-free survival rates [16].

The study aimed to analyse the impact of different molecular subtypes of BC on the rate of locoregional recurrence in our centre and correlate this with the age of the patients.

## Materials and methods

This is a retrospective study that included all female patients who had surgery (mastectomy or BCS) for BC (stage Ia to stage IIIc) in our centre and later developed locoregional recurrence during the study period (January 1, 2012, to December 31, 2014). Patients with metastatic disease and male patients were excluded.

Data were retrieved from the pathology department and the patients’ files were reviewed to assess the primary BC molecular profile. Some of the pathology reports reviewed did not have complete molecular profiling. The tissue blocks were retrieved, and molecular profiling was completed by the pathology department. The patients were divided into two groups: group I (<40 years old) and Group II (≥40 years old). 

The molecular classification was based on estrogen receptor (ER), progesterone receptor (PR), Ki-67 proliferation index, and human epidermal growth factor receptor 2 (HER2)/neu receptor status on the paraffin blocks. The luminal classification was recorded as positive (whether it was weakly or strongly expressed) or negative. The Her-2/neu was recorded as positive or negative in all the patients and the Ki-67 proliferation index was recorded as high (15% or higher) or low (less than 15%).

## Results

During the study period, 206 patients developed locoregional recurrence after BC surgery. In 22 patients the pathology was cystosarcoma phyllodes, therefore these patients were not included in further analysis, leaving 184 patients. Group I included 60 patients (32.6%), while Group II included 124 patients (67.4%).

Axillary lymph nodes were positive in 48 patients (80%) in group I and in 102 patients (82.3%) in group II.

The different histopathological types across the two groups are outlined in Table 1.

**Table 1:** The histopathology in both groups

Histopathology	Group I		Group II		Total	
	No.	%	No.	%	No.	%
Invasive duct carcinoma (IDC)	54	90.1	100	80.6	154	83.7
Invasive Lobular carcinoma (ILC)	0	0	14	11.3	14	7.6
Mixed IDC & ILC	0	0	8	6.5	8	4.3
Mucoid Carcinoma	2	3.3	2	1.6	4	2.2
Atypical Medullary Carcinoma	2	3.3	0	0	2	1.1
Cribriform Carcinoma	2	3.3	0	0	2	1.1
Total	60	100	124	100	184	100

In group I, the initial operation was BCS in 44 patients (73.3%), while MRM was done in 16 patients (26.7%). In group II, BCS was done in 60 patients (48.4%) while MRM was done in 64 patients (51.6%).

In group I, 12 patients (20%) received adjuvant radiotherapy (after BCS). Thirty-two patients (53.3%) received adjuvant radio-chemotherapy and 10 patients (22.9%) received neoadjuvant chemotherapy. Thirty-eight patients (63.3%) of this group were on hormonal therapy.

In group II, 20 patients (16.1%) received adjuvant radiotherapy, and 40 patients (32.2%) received adjuvant radio-chemotherapy (after BCS). Twenty-eight patients (22.6%) in this group received neoadjuvant chemotherapy. Hormonal treatment was the only adjuvant therapy in 16 patients (12.9%). However, hormonal therapy was given to 41 patients (66.1%) in total in this group.

In group I, recurrence occurred after a mean of 4.3 years (range 1 to 23). While in group II, the mean onset of recurrence was 3.8 years (range 1 to 14).

In group I, 20 patients (33.3%) were luminal positive, more than half of them (12 patients) were strong positive [+++], while it was negative in 40 patients (66.7%). In group II, 44 patients were luminal positive (35.5%), the majority, 32 patients (25.8%) had strong positive [+++] luminal pattern, while in 80 patients (64.5%) of this group, the luminal pattern was negative.

The status of Her2/neu receptor in both groups is displayed in Table 2. The status of Ki-67 proliferation index is displayed in Table 3 and the molecular subtypes are summarized in Table 4.

**Table 2:** The status of Her-2/neu receptor in both groups.

Her-2/neu receptor	Group I		Group II		Total	
	No.	%	No.	%	No.	%
Positive	36	60	76	61.3	102	60.9
Negative	24	40	48	38.7	72	39.1
Total	60	100	124	100	184	100

**Table 3:** The incidence of high and low Ki-67 proliferation index in both groups.

Ki-67 proliferation index	Group I		Group II		Total	
	No.	%	No.	%	No.	%
High	42	70	94	75.8	136	73.9
Low	18	30	30	24.1	48	26.1
Total	60	100	124	100	184	100

**Table 4:** The incidence of different breast cancer subtypes in both groups

Breast cancer Subtype	Group I		Group II		Total	
	No.	%	No.	%	No.	%
Luminal A subtype	12	20	32	25.8	44	23.9
Luminal B subtype	8	13.3	12	9.7	20	10.9
Her-2/neu enriched subtype	18	30	38	30.6	56	30.4
Triple-negative subtype	22	36.7	42	33.9	64	34.8
Total	60	100	124	100	184	100

## Discussion

BC is an age-related disease. Advancing age is considered the second most significant risk factor for BC. Age interactions are frequently reported in studies that examine aetiology, prognosis, and treatment [17]. 

The study by Alieldin et al. which included a series of 941 patients with BC, showed that 19.2% of them were below 40 years, and 80.8% were 40 years or more [18]. Similarly, in the study done by Schlichting et al., which was a comparative study, showed that the incidence of patients diagnosed with BC below the age of 40 years in Egypt was low (18.1%) [19].

López et al. had estimated remaining free of local recurrence probability five and 10 years after primary treatment to be 97.7% and 94.5% respectively. Age had a significant impact in predicting local relapse on multivariate analysis. For patients ≤40 years old, the relative risk of locoregional recurrence was 5.27 and 3.7 with regards to patients aged 41-50 years and patients older than 50 years old, respectively. They concluded that patients ≤40 years old have a higher risk of local failure than older patients; factors other than age, for instance, close margins, intraductal component, and size of tumour can be masked by the effects of a higher dose of radiotherapy [13].

Matthews et al. reported a crude local recurrence rate of 67% for patients aged 20-29 years and 41% for patients aged 30-39 years in an early radical mastectomy series, whereas in women aged 40 years, local failure rates were 21% to 25% [20]. The same crude rate 67% for ages 20-29 years was documented by Donegan et al. in their series of radical mastectomy, while it was 46% for ages 30-39 years, compared with <25% for those >40 years old [21]. Fowble et al. previously had more or less similar results as well [22].

The axillary lymph nodes were positive in most of the recurrent cases, (80% in group I and 82.3% in group II). This goes with the work of Millar et al. who demonstrated that the local failure has been associated with young age (<50 years), tumour size (>T2), negative hormone receptor status, and lymph node involvement. Similar reports were recorded by Arriagada et al. and Anders et al. [10,23-24].

Infiltrating duct carcinoma (IDC) is considered by Lopez et al. to be one of the risk factors for recurrence. This is clear in our results, where IDC was the main histopathology (90.1% for group I and 80.6% for group II) [13].

In group I (<40 years), recurrence was more common after BCS (73.3%) than after MRM (26.7%). In contrast, group II recurrence rates after BCS and MRM were almost similar (48.4% vs 51.6% respectively). Patients in this study had a recurrence of their disease after a mean of two and three years in groups I and II respectively. This is similar to the work of Millar et al. whose mean time to local recurrence was (2.8 vs 4.2 years) [10].

Neguyen et al. mentioned that gene expression can have a clinical impact and can be used to separate patients according to tumour morphology which can have different effects. Subtypes of basal and HER-2/neu are more likely to be grade 3 than the subtype luminal A, which is usually graded 2 or lower. They reported better prognosis with the luminal A subtype, though significantly poorer recurrence rates and overall survival was observed for the HER-2/neu and basal subgroups [11].

Brenton et al., Carey et al., and Neguyen et al. reported that the pattern of recurrence, whether local or distant, can differ according to the BC subtypes (ER, PR, and HER-2/neu) in patients who receive breast-conserving treatment (BCT). They observed that the overall five-year local recurrence rate of 1.8% after BCT varied by subtype. On multivariant analysis with luminal A as a baseline, local recurrence was significantly higher with the HER-2/neu and basal subtypes, while margin status, age, size of the tumour, and nodal status were not significant [11,25-26].

A study by Haffty et al. included 482 females who underwent BCT. They documented no significant difference in the ipsilateral breast relapse-free five-year survival between triple-negative patients and the rest of the cohort (83% vs 83%). However, the isolated regional nodal recurrence-free survival was significantly lower in the triple-negative patients (94%) compared to the rest of the cohort (99%) [10]. Additionally, triple-negative cancers had comparable average local recurrence rates in a report by Dent et al. to those in the non-triple-negative category (13% vs 12%), but the meantime to local recurrence was substantially shorter for the triple-negative patients compared with the other BC subtypes (2.8 vs 4.2 years) [27].

In this series, we found that among our recurrent cases, the luminal negative subtype was more prevalent in both groups (66.7% and 64.5% respectively). Luminal A reported cases were higher in group II (25.8%) than group I (20%). While the Luminal B reported cases were higher in group I (13.3% vs 9.7% respectively). In these recurrent cases, the incidence of the Her-2/neu enriched subtype was similar in both groups. Triple-negative patients in this study were prominent in both groups (36.7% and 33.9% respectively).

The disparity in the results between different studies and our study may be attributed to different study populations with different characteristics. We observed a steady reduction in locoregional recurrence rates over the study period, and this is possibly routed to better imaging techniques, focusing on achieving negative margins and more effective adjuvant systemic treatment regimens. This was particularly evident in patients with luminal or hormone-responsive cancers, which represented more than a third of our sample. The findings of this study indicate that subtype recognition using ER, PR, and HER-2/neu immunophenotypes is helpful in estimating the probability of locoregional recurrence after mastectomy. However, the causes of locoregional recurrence for all subtypes were multifactorial.

The limitations of this research include being a retrospective designed study and small sample size. In this study, we did not include all breast cancer patients, so the incidence of recurrence rates could not be commented on. Additionally, few patients did not complete their adjuvant treatment rendering it a suboptimal treatment.

## Conclusions

To conclude, in our series, the most common molecular subtype found in the recurrent cases was the luminal negative subtypes, with a relatively similar pattern across both age groups. The results of this study can be used as a basis for large prospective studies in our centre to further analyse the effect of molecular subtyping on the recurrence rates of BC.
